# Re-refinement of sodium ammonium sulfate dihydrate at 170 K

**DOI:** 10.1107/S2414314620012754

**Published:** 2020-09-25

**Authors:** Tamira Eckhardt, Christoph Wagner, Peter Imming, Rüdiger W. Seidel

**Affiliations:** a Institut für Pharmazie, Wolfgang-Langenbeck-Str. 4, 06120 Halle (Saale), Germany; b Institut für Chemie, Kurt-Mothes-Str. 2, 06120 Halle (Saale), Germany; Vienna University of Technology, Austria

**Keywords:** crystal structure, hydrogen bonding, chirality, chain structure, ferroelectric, sulfate mineral

## Abstract

The title compound, sodium ammonium sulfate dihydrate (SASD), NaNH_4_SO_4_·2H_2_O, a synthetic analogue of the mineral lecontite, is a well known ferroelectric. The crystal structure of the paraelectric phase has been re-refined at 170 K on the basis of single-crystal X-ray data, improving the previous study [Arzt & Glazer (1994 ▸). *Acta Cryst.* B**50**, 425–431] in terms of accuracy regarding hydrogen-atom positions and thus details of the hydrogen bonding. O—H⋯O and N—H⋯O hydrogen bonds between the principal building units [Na(OH_2_)_4_O_2_ octa­hedra, SO_4_ tetra­hedra and ammonium cations] constitute a three-dimensional network structure.

## Abstract 2

The crystal structure of sodium ammonium sulfate dihydrate has been redetermined at 170 K on the basis of single-crystal X-ray data.

## Graphical abstract

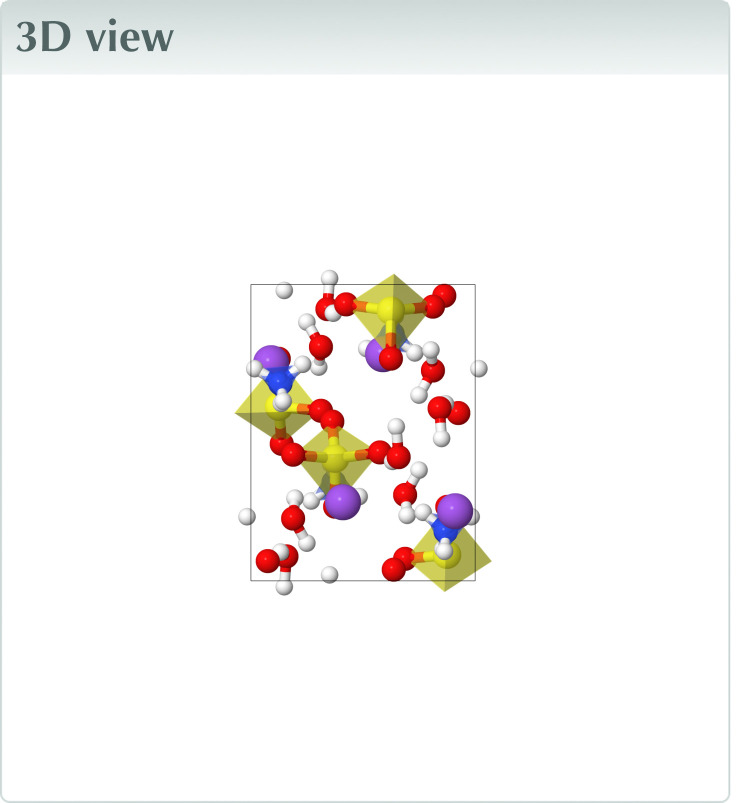

## Structure description

The title compound, sodium ammonium sulfate dihydrate (SASD), NaNH_4_SO_4_·2H_2_O, is the synthetic analogue of the mineral lecontite (Hawthorne *et al.*, 2000 ▸), as revealed by Faust & Bloss (1963 ▸) through a diffractometry study of both synthetic and natural material. The crystal structure of SASD was first determined by Corazza *et al.* (1967 ▸) from equi-inclination Weissenberg photographs at room temperature. Arzt & Glazer (1994 ▸) redetermined the crystal structure at room temperature based on serial detector data. Properties of the well-known ferroelectric SASD have been widely studied (Arzt & Glazer, 1994 ▸; Fawcett *et al.*, 1975 ▸; Genin & O’Reilly, 1969 ▸; Hilczer *et al.*, 1991 ▸, 1992 ▸, 1993 ▸; Kanesaka & Ozaki, 1994 ▸; Kassem & Hedewy, 1988 ▸; Kloprogge *et al.*, 2006 ▸; Lipinski *et al.*, 2003 ▸; Lipinski & Kuriata, 2005 ▸; Ono *et al.*, 1993 ▸; Osaka, 1978 ▸; Osaka & Makita, 1970 ▸; Ribeiro *et al.*, 2006 ▸). Kloprogge *et al.* (2006 ▸) also reported a Rietveld refinement of the structure of SASD at room temperature, thereby confirming the results of the previous single-crystal X-ray analyses. We have now re-refined the crystal structure of the paralectric phase of SASD at 170 K on the basis of single-crystal X-ray diffraction data.

**Figure 1:**
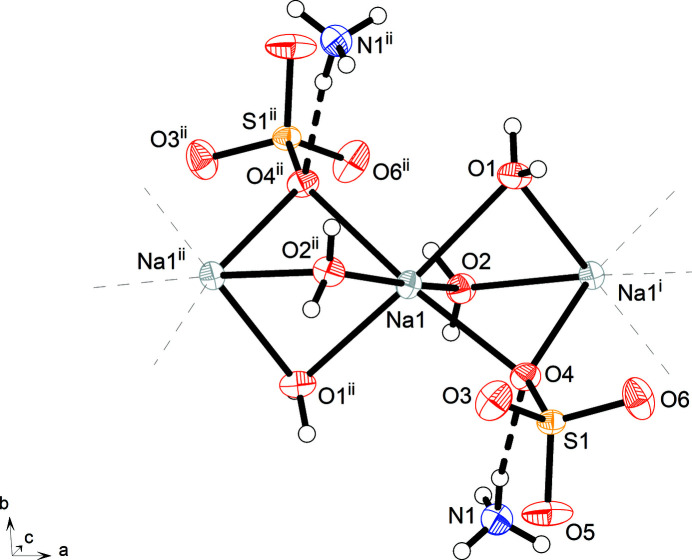
Section of the crystal structure of SASD, viewed approximately along the *c-*axis direction towards the origin. Displacement ellipsoids are drawn at the 50% probability level. Hydrogen atoms are represented by small spheres of arbitrary radius. Thick dashed lines represent hydrogen bonds. [Symmetry codes: (i) *x* + 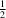 , −*y* + 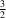 , −*z* + 1; (ii) *x* − 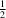 , −*y* + 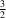 , −*z* + 1.]

**Figure 2:**
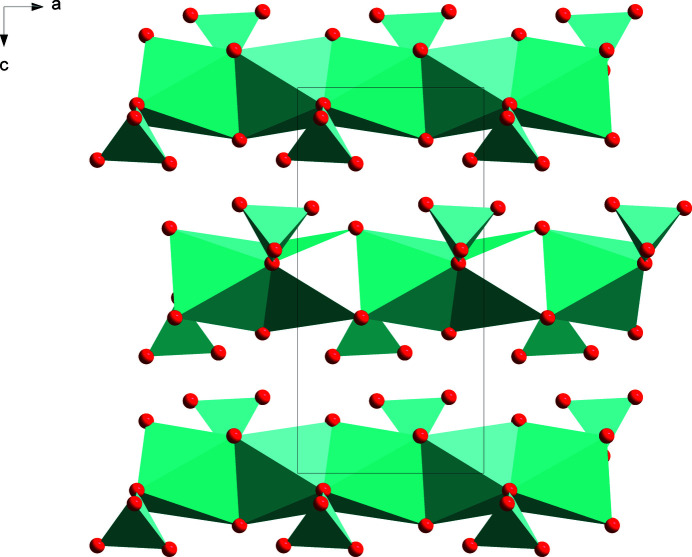
The crystal structure of SASD viewed down the *b*-axis direction, showing the chains featuring face-sharing NaO_6_ octa­hedra with appended SO_4_ tetra­hedra. Ammonium ions are omitted for clarity.

As shown in Fig. 1 ▸, the sodium cation is hexa-coordinated with a considerably distorted octa­hedral coordination sphere formed by four water mol­ecules in the equatorial plane and two sulfate O atoms in the apical positions. Selected bond lengths and angles are listed in Table 1 ▸. Each of the ligands links two sodium cations in a *μ*-coordination mode, resulting in chains along the [100] direction with the Na cations located near to a 2_1_ screw axis. Na1⋯Na1^i^ and Na1⋯Na1^ii^ are separated by 3.1317 (2) and 3.1316 (2) Å, respectively [symmetry codes: (i) *x* − 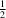 , −*y* + 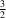 , −*z* + 1; (ii) *x* + 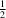 , −*y* + 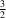 , −*z* + 1]. The chains can be described as consisting of NaO_6_ octa­hedra sharing one face (Fig. 2 ▸) defined by atoms O1, O2 and O4. The sulfate anion exhibits the typical tetra­hedral shape with an r.m.s. deviation from exact *T*_d_ symmetry of only 0.0092 Å, as calculated with MOLSYM in *PLATON* (Spek, 2020 ▸). In the chains, the SO_4_ tetra­hedra have one O atom in common with a pair of NaO_6_ octa­hedra. Chain motifs are encountered in the structures of many other sulfates (Gorogotskaya & Bokii, 1973 ▸).

The crystal structure features hydrogen bonds of the O—H⋯O and N—H⋯O type (Table 2 ▸). The water mol­ecules form medium–strong and nearly linear intra- and inter­chain hydrogen bonds to sulfate oxygen atoms. The inter­stices between the [Na(*μ*-SO_4_)(*μ*-H_2_O)_2_]_*n*_^−^ chains accommodate the ammonium cations, which form hydrogen bonds to sulfate oxygen atoms, thus establishing a three-dimensional network. The positions of the ammonium hydrogen atoms determined in the current study appear to be more accurate than those in the room-temperature structure reported by Arzt & Glazer (1994 ▸). Note that details of hydrogen bonding were not discussed in the latter report; based on the reported structure data (Arzt & Glazer, 1994 ▸), N—H distances range between 0.73 and 0.99 Å. Corazza *et al.* (1967 ▸) did not refine hydrogen-atom parameters in the original room-temperature structure determination but included their presumed positions in the structure-factor calculation for the final refinement of the non-hydrogen atoms. In the current study, semi-free refinement applying only similarity restraints on the 1,2-distances involving hydrogen atoms resulted in reasonable hydrogen-atom parameters and a sensible hydrogen-bonding scheme.

The absolute structure of the crystal was established by anomalous-dispersion effects in the diffraction data, as indicated by a Flack *x* parameter close to zero with a reasonably small standard uncertainty (Table 3 ▸). The Hooft *y* parameter (Hooft *et al.*, 2008 ▸), as calculated with *PLATON*, is 0.07 (2). Inter­estingly, the structure exhibits chirality opposite to the previously reported room temperature structures (Corazza *et al.*, 1967 ▸; Arzt & Glazer, 1994 ▸).

## Synthesis and crystallization

A crystal of the title compound suitable for single-crystal X-ray analysis was obtained unintentionally from a solution in an aceto­nitrile/water mixture after synthesis of an organic compound. Ammonium ions and sodium sulfate in this mixture originated from an employed reagent and the drying agent, respectively.

## Refinement

Crystal data, data collection and structure refinement details are summarized in Table 3 ▸.

## Supplementary Material

Crystal structure: contains datablock(s) global, I. DOI: 10.1107/S2414314620012754/wm4138sup1.cif

Structure factors: contains datablock(s) I. DOI: 10.1107/S2414314620012754/wm4138Isup2.hkl

Supporting information file. DOI: 10.1107/S2414314620012754/wm4138Isup3.cml

CCDC reference: 2032745

Additional supporting information:  crystallographic information; 3D view; checkCIF report

## Figures and Tables

**Table 1:** Selected geometric parameters (Å, °)

Na1—O2^i^	2.3229 (15)	Na1—O4^i^	2.4546 (14)
Na1—O4	2.3733 (14)	S1—O5	1.4628 (14)
Na1—O2	2.4054 (16)	S1—O4	1.4721 (12)
Na1—O1	2.4087 (15)	S1—O3	1.4728 (15)
Na1—O1^i^	2.4389 (16)	S1—O6	1.4740 (15)
			
O2^i^—Na1—O4	111.05 (5)	O4—Na1—O4^i^	167.55 (5)
O2^i^—Na1—O2	166.61 (6)	O2—Na1—O4^i^	86.58 (5)
O4—Na1—O2	81.31 (5)	O1—Na1—O4^i^	92.00 (5)
O2^i^—Na1—O1	103.97 (6)	O1^i^—Na1—O4^i^	81.23 (5)
O4—Na1—O1	83.54 (5)	O5—S1—O4	109.42 (8)
O2—Na1—O1	81.97 (5)	O5—S1—O3	109.02 (11)
O2^i^—Na1—O1^i^	83.03 (5)	O4—S1—O3	110.07 (8)
O4—Na1—O1^i^	101.42 (5)	O5—S1—O6	109.17 (11)
O2—Na1—O1^i^	89.58 (5)	O4—S1—O6	109.83 (8)
O1—Na1—O1^i^	169.50 (3)	O3—S1—O6	109.31 (9)
O2^i^—Na1—O4^i^	81.28 (5)		

Symmetry code: (i) 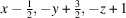 .

**Table 2:** Hydrogen-bond geometry (Å, °)

*D*—H⋯*A*	*D*—H	H⋯*A*	*D*⋯*A*	*D*—H⋯*A*
O1—H1*A*⋯O6^ii^	0.84 (2)	1.99 (2)	2.812 (2)	165 (3)
O1—H1*B*⋯O5^iii^	0.85 (2)	1.89 (2)	2.7439 (19)	175 (3)
O2—H2*A*⋯O3^iv^	0.81 (2)	1.98 (2)	2.781 (2)	171 (3)
O2—H2*B*⋯O6^i^	0.81 (2)	1.98 (2)	2.781 (2)	175 (3)
N1—H1*C*⋯O4	0.81 (2)	2.14 (2)	2.948 (2)	172 (3)
N1—H1*D*⋯O3^iv^	0.83 (2)	2.03 (2)	2.854 (2)	172 (3)
N1—H1*E*⋯O3^v^	0.81 (2)	2.23 (2)	2.977 (2)	152 (3)
N1—H1*E*⋯O5^v^	0.81 (2)	2.60 (3)	3.324 (3)	149 (3)
N1—H1*F*⋯O5^vi^	0.81 (2)	2.58 (3)	3.245 (3)	140 (3)
N1—H1*F*⋯O6^vi^	0.81 (2)	2.13 (2)	2.897 (3)	157 (3)

Symmetry codes: (i) 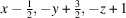 ; (ii) 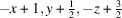 ; (iii) 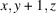 ; (iv) 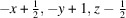 ; (v) 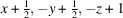 ; (vi) 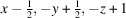 .

**Table 3:** Experimental details

Crystal data	
Chemical formula	NaNH_4_SO_4_·2H_2_O
*M*_r_	173.12
Crystal system, space group	Orthorhombic, *P*2_1_2_1_2_1_
Temperature (K)	170
*a*, *b*, *c* (Å)	6.2001 (2), 8.1917 (3), 12.8121 (6)
*V* (Å^3^)	650.72 (4)
*Z*	4
Radiation type	Mo *K*α
μ (mm^−1^)	0.53
Crystal size (mm)	0.50 × 0.48 × 0.28

Data collection	
Diffractometer	Stoe IPDS 2T
Absorption correction	Multi-scan [*MULABS* (Blessing, 1995 ▸) in *PLATON* (Spek, 2020 ▸)]
*T*_min_, *T*_max_	0.728, 1.117
No. of measured, independent and observed [*I* > 2σ(*I*)] reflections	14489, 1754, 1690
*R*_int_	0.038

Refinement	
*R*[*F* ^2^ > 2σ(*F* ^2^)], *wR*(*F* ^2^), *S*	0.023, 0.058, 1.09
No. of reflections	1754
No. of parameters	114
No. of restraints	12
H-atom treatment	All H-atom parameters refined
Δρ_max_, Δρ_min_ (e Å^−3^)	0.20, −0.34
Absolute structure	Flack *x* determined using 672 quotients [(*I* ^+^)−(*I* ^−^)]/[(*I* ^+^)+(*I* ^−^)] (Parsons *et al.*, 2013 ▸)
Absolute structure parameter	−0.05 (2)

Computer programs: *X-AREA* and *X-RED* (Stoe & Cie, 2016 ▸), *SHELXT* (Sheldrick, 2015*a* ▸), *SHELXL2018* (Sheldrick, 2015*b* ▸), *DIAMOND* (Brandenburg, 2018 ▸), *enCIFer* (Allen *et al.*, 2004 ▸) and *publCIF* (Westrip, 2010 ▸).

## full crystallographic data

### Crystal data

**Table:**

Na^+^·NH_4_^+^·SO_4_^2^^−^·2H_2_O	*D*_x_ = 1.767 Mg m^−^^3^
*M**_r_* = 173.12	Mo *K*α radiation, λ = 0.71073 Å
Orthorhombic, *P*2_1_2_1_2_1_	Cell parameters from 15574 reflections
*a* = 6.2001 (2) Å	θ = 3.0–29.5°
*b* = 8.1917 (3) Å	µ = 0.53 mm^−^^1^
*c* = 12.8121 (6) Å	*T* = 170 K
*V* = 650.72 (4) Å^3^	Prism, colourless
*Z* = 4	0.50 × 0.48 × 0.28 mm
*F*(000) = 360	

### Data collection

**Table:**

STOE IPDS 2T diffractometer	1754 independent reflections
Radiation source: sealed X-ray tube, Incoatec Iµs	1690 reflections with *I* > 2σ(*I*)
Detector resolution: 6.67 pixels mm^-1^	*R*_int_ = 0.038
ω scans	θ_max_ = 29.2°, θ_min_ = 3.0°
Absorption correction: multi-scan [*MULABS* (Blessing, 1995) in *PLATON* (Spek, 2020)]	*h* = −8→7
*T*_min_ = 0.728, *T*_max_ = 1.117	*k* = −11→11
14489 measured reflections	*l* = −17→17

### Refinement

**Table:**

Refinement on *F*^2^	Secondary atom site location: difference Fourier map
Least-squares matrix: full	Hydrogen site location: difference Fourier map
*R*[*F*^2^ > 2σ(*F*^2^)] = 0.023	All H-atom parameters refined
*wR*(*F*^2^) = 0.058	*w* = 1/[σ^2^(*F*_o_^2^) + (0.0357*P*)^2^ + 0.0942*P*] where *P* = (*F*_o_^2^ + 2*F*_c_^2^)/3
*S* = 1.09	(Δ/σ)_max_ < 0.001
1754 reflections	Δρ_max_ = 0.20 e Å^−^^3^
114 parameters	Δρ_min_ = −0.34 e Å^−^^3^
12 restraints	Absolute structure: Flack *x* determined using 672 quotients [(*I*^+^)-(*I*^-^)]/[(*I*^+^)+(*I*^-^)] (Parsons *et al.*, 2013)
Primary atom site location: dual	Absolute structure parameter: −0.05 (2)

### Special details

**Table:**

Geometry. All esds (except the esd in the dihedral angle between two l.s. planes) are estimated using the full covariance matrix. The cell esds are taken into account individually in the estimation of esds in distances, angles and torsion angles; correlations between esds in cell parameters are only used when they are defined by crystal symmetry. An approximate (isotropic) treatment of cell esds is used for estimating esds involving l.s. planes.
Refinement. Hydrogen-atom positions were located in a difference Fourier map, and their parameters were refined with standard similarity restraints on 1,2-distances for O—H and and N—H bonds (with a standard uncertainty of 0.02 Å). *U*_iso_(H) values were refined freely.

### Fractional atomic coordinates and isotropic or equivalent isotropic displacement parameters (Å^2^)

**Table:**

	*x*	*y*	*z*	*U*_iso_*/*U*_eq_	
Na1	0.08933 (10)	0.73476 (8)	0.51432 (5)	0.01968 (17)	
S1	0.37520 (7)	0.41221 (5)	0.62681 (3)	0.01762 (11)	
O1	0.3414 (2)	0.91744 (17)	0.59691 (11)	0.0248 (3)	
H1A	0.368 (6)	0.903 (4)	0.6609 (18)	0.060 (10)*	
H1B	0.351 (5)	1.020 (3)	0.587 (2)	0.050 (9)*	
O2	0.3121 (2)	0.79007 (17)	0.36474 (11)	0.0228 (3)	
H2A	0.303 (5)	0.721 (3)	0.320 (2)	0.040 (8)*	
H2B	0.250 (5)	0.873 (3)	0.348 (2)	0.035 (7)*	
O3	0.1880 (3)	0.4256 (2)	0.69708 (12)	0.0326 (3)	
O4	0.3627 (2)	0.53757 (14)	0.54471 (10)	0.0212 (3)	
O5	0.3756 (4)	0.25020 (16)	0.57880 (12)	0.0390 (4)	
O6	0.5751 (2)	0.4335 (2)	0.68758 (12)	0.0337 (4)	
N1	0.3688 (3)	0.3331 (2)	0.35538 (13)	0.0244 (3)	
H1C	0.356 (5)	0.394 (3)	0.4051 (19)	0.042 (8)*	
H1D	0.364 (6)	0.401 (3)	0.308 (2)	0.051 (9)*	
H1E	0.483 (4)	0.284 (4)	0.354 (3)	0.056 (10)*	
H1F	0.269 (4)	0.269 (3)	0.358 (3)	0.050 (9)*	

### Atomic displacement parameters (Å^2^)

**Table:**

	*U*^11^	*U*^22^	*U*^33^	*U*^12^	*U*^13^	*U*^23^
Na1	0.0170 (3)	0.0211 (3)	0.0209 (3)	0.0009 (2)	0.0010 (3)	−0.0001 (2)
S1	0.02186 (19)	0.01448 (17)	0.01652 (18)	−0.00021 (15)	0.00033 (15)	0.00224 (14)
O1	0.0334 (7)	0.0183 (6)	0.0226 (6)	−0.0006 (6)	−0.0037 (5)	−0.0011 (5)
O2	0.0253 (6)	0.0237 (6)	0.0195 (6)	0.0023 (5)	−0.0034 (5)	0.0005 (5)
O3	0.0308 (7)	0.0383 (8)	0.0288 (7)	0.0050 (6)	0.0113 (6)	0.0072 (6)
O4	0.0259 (6)	0.0170 (5)	0.0206 (5)	−0.0001 (5)	−0.0020 (5)	0.0056 (4)
O5	0.0715 (11)	0.0150 (6)	0.0304 (7)	0.0014 (8)	0.0006 (8)	−0.0004 (5)
O6	0.0311 (7)	0.0402 (8)	0.0298 (7)	−0.0088 (6)	−0.0106 (6)	0.0138 (6)
N1	0.0263 (8)	0.0266 (7)	0.0201 (7)	0.0006 (7)	0.0007 (7)	−0.0009 (6)

### Geometric parameters (Å, º)

**Table:**

Na1—O2^i^	2.3229 (15)	S1—O3	1.4728 (15)
Na1—O4	2.3733 (14)	S1—O6	1.4740 (15)
Na1—O2	2.4054 (16)	O1—H1A	0.84 (2)
Na1—O1	2.4087 (15)	O1—H1B	0.85 (2)
Na1—O1^i^	2.4389 (16)	O2—H2A	0.81 (2)
Na1—O4^i^	2.4546 (14)	O2—H2B	0.81 (2)
Na1—Na1^ii^	3.1316 (2)	N1—H1C	0.814 (19)
Na1—Na1^i^	3.1317 (2)	N1—H1D	0.828 (19)
Na1—H2B	2.61 (3)	N1—H1E	0.81 (2)
S1—O5	1.4628 (14)	N1—H1F	0.81 (2)
S1—O4	1.4721 (12)		
			
O2^i^—Na1—O4	111.05 (5)	O1^i^—Na1—H2B	89.2 (6)
O2^i^—Na1—O2	166.61 (6)	O4^i^—Na1—H2B	68.8 (5)
O4—Na1—O2	81.31 (5)	Na1^ii^—Na1—H2B	59.5 (6)
O2^i^—Na1—O1	103.97 (6)	Na1^i^—Na1—H2B	104.3 (6)
O4—Na1—O1	83.54 (5)	O5—S1—O4	109.42 (8)
O2—Na1—O1	81.97 (5)	O5—S1—O3	109.02 (11)
O2^i^—Na1—O1^i^	83.03 (5)	O4—S1—O3	110.07 (8)
O4—Na1—O1^i^	101.42 (5)	O5—S1—O6	109.17 (11)
O2—Na1—O1^i^	89.58 (5)	O4—S1—O6	109.83 (8)
O1—Na1—O1^i^	169.50 (3)	O3—S1—O6	109.31 (9)
O2^i^—Na1—O4^i^	81.28 (5)	Na1—O1—Na1^ii^	80.48 (4)
O4—Na1—O4^i^	167.55 (5)	Na1—O1—H1A	118 (2)
O2—Na1—O4^i^	86.58 (5)	Na1^ii^—O1—H1A	112 (2)
O1—Na1—O4^i^	92.00 (5)	Na1—O1—H1B	127 (2)
O1^i^—Na1—O4^i^	81.23 (5)	Na1^ii^—O1—H1B	112 (2)
O2^i^—Na1—Na1^ii^	144.84 (5)	H1A—O1—H1B	105 (3)
O4—Na1—Na1^ii^	50.70 (4)	Na1^ii^—O2—Na1	82.93 (4)
O2—Na1—Na1^ii^	47.40 (4)	Na1^ii^—O2—H2A	118 (2)
O1—Na1—Na1^ii^	50.18 (4)	Na1—O2—H2A	113 (2)
O1^i^—Na1—Na1^ii^	126.60 (5)	Na1^ii^—O2—H2B	127 (2)
O4^i^—Na1—Na1^ii^	118.04 (5)	Na1—O2—H2B	95 (2)
O2^i^—Na1—Na1^i^	49.66 (4)	H2A—O2—H2B	111 (3)
O4—Na1—Na1^i^	141.29 (5)	S1—O4—Na1	129.03 (8)
O2—Na1—Na1^i^	117.40 (5)	S1—O4—Na1^ii^	136.05 (8)
O1—Na1—Na1^i^	130.11 (5)	Na1—O4—Na1^ii^	80.86 (4)
O1^i^—Na1—Na1^i^	49.34 (4)	H1C—N1—H1D	99 (3)
O4^i^—Na1—Na1^i^	48.44 (4)	H1C—N1—H1E	114 (3)
Na1^ii^—Na1—Na1^i^	163.71 (5)	H1D—N1—H1E	110 (3)
O2^i^—Na1—H2B	149.9 (5)	H1C—N1—H1F	107 (3)
O4—Na1—H2B	99.0 (5)	H1D—N1—H1F	116 (3)
O2—Na1—H2B	18.0 (5)	H1E—N1—H1F	110 (3)
O1—Na1—H2B	80.9 (6)		

Symmetry codes: (i) *x*−1/2, −*y*+3/2, −*z*+1; (ii) *x*+1/2, −*y*+3/2, −*z*+1.

### Hydrogen-bond geometry (Å, º)

**Table:**

*D*—H···*A*	*D*—H	H···*A*	*D*···*A*	*D*—H···*A*
O1—H1*A*···O6^iii^	0.84 (2)	1.99 (2)	2.812 (2)	165 (3)
O1—H1*B*···O5^iv^	0.85 (2)	1.89 (2)	2.7439 (19)	175 (3)
O2—H2*A*···O3^v^	0.81 (2)	1.98 (2)	2.781 (2)	171 (3)
O2—H2*B*···O6^i^	0.81 (2)	1.98 (2)	2.781 (2)	175 (3)
N1—H1*C*···O4	0.81 (2)	2.14 (2)	2.948 (2)	172 (3)
N1—H1*D*···O3^v^	0.83 (2)	2.03 (2)	2.854 (2)	172 (3)
N1—H1*E*···O3^vi^	0.81 (2)	2.23 (2)	2.977 (2)	152 (3)
N1—H1*E*···O5^vi^	0.81 (2)	2.60 (3)	3.324 (3)	149 (3)
N1—H1*F*···O5^vii^	0.81 (2)	2.58 (3)	3.245 (3)	140 (3)
N1—H1*F*···O6^vii^	0.81 (2)	2.13 (2)	2.897 (3)	157 (3)

Symmetry codes: (i) *x*−1/2, −*y*+3/2, −*z*+1; (iii) −*x*+1, *y*+1/2, −*z*+3/2; (iv) *x*, *y*+1, *z*; (v) −*x*+1/2, −*y*+1, *z*−1/2; (vi) *x*+1/2, −*y*+1/2, −*z*+1; (vii) *x*−1/2, −*y*+1/2, −*z*+1.
